# Subspace Identification and Classification of Healthy Human Gait

**DOI:** 10.1371/journal.pone.0065063

**Published:** 2013-07-08

**Authors:** Vinzenz von Tscharner, Hendrik Enders, Christian Maurer

**Affiliations:** Human Performance Laboratory, Faculty of Kinesiology, University of Calgary, Calgary, AB, Canada; University of California, Merced, United States of America

## Abstract

**Purpose:**

The classification between different gait patterns is a frequent task in gait assessment. The base vectors were usually found using principal component analysis (PCA) is replaced by an iterative application of the support vector machine (SVM). The aim was to use classifyability instead of variability to build a subspace (SVM space) that contains the information about classifiable aspects of a movement. The first discriminant of the SVM space will be compared to a discriminant found by an independent component analysis (ICA) in the SVM space.

**Methods:**

Eleven runners ran using shoes with different midsoles. Kinematic data, representing the movements during stance phase when wearing the two shoes, was used as input to a PCA and SVM. The data space was decomposed by an iterative application of the SVM into orthogonal discriminants that were able to classify the two movements. The orthogonal discriminants spanned a subspace, the SVM space. It represents the part of the movement that allowed classifying the two conditions. The data in the SVM space was reconstructed for a visual assessment of the movement difference. An ICA was applied to the data in the SVM space to obtain a single discriminant. Cohen's d effect size was used to rank the PCA vectors that could be used to classify the data, the first SVM discriminant or the ICA discriminant.

**Results:**

The SVM base contains all the information that discriminates the movement of the two shod conditions. It was shown that the SVM base contains some redundancy and a single ICA discriminant was found by applying an ICA in the SVM space.

**Conclusions:**

A combination of PCA, SVM and ICA is best suited to extract all parts of the gait pattern that discriminates between the two movements and to find a discriminant for the classification of dichotomous kinematic data.

## Introduction

Differences of human movements that are caused by various gait abnormalities or footwear conditions are in general obscured by a large amplitude of the overall movement, and by a high intra- and inter-subject variability [1], [2]. Footwear interventions introduce only small changes compared with the variability due to the movement itself [3], [4].

Vector-based pattern recognition methods such as principal component analysis (PCA) or support vector machines (SVM) and independent component analysis (ICA) have become promising tools for analyzing human movement [5]–[10]. Compared to the traditional approach where discrete kinematic variables were pre-selected intuitively by the researcher, pattern recognition methods allow analyzing all of the information within a set of kinematic data. PCA was applied to kinematic data sets in order to study human movement with the purpose to identify movement features that are sensitive to gender, age, speed, footwear or pathological conditions [3], [4], [11]–[13].

A time series of length *N* can be represented as a vector with *N* components in an *N* dimensional vector space. The time-base (axes) consists of vectors where all but one component are zeros and the component representing the time, *t*, is one. If multiple time series exist, their vectors span the vector space; we call it the data space. As an example, kinematic data sets consist of time series indicating the positions of markers attached to the body. However, beside the time-base, which allows us to follow the temporal aspects of the marker positions, other bases can be computed which allow observing specific aspects of the data. The most widely used base for time series consists of sine and cosine waves and the data are represented by the Fourier coefficients. The choice of the base that allows the researcher to visualize specific movement aspects depends on the research question and is a crucial factor as it is sensitive to whether the base axes distribute the data with respect to variability [14], separability, classifyability or independence.

To study and visualize the pattern of a movement a PCA is most useful. The PCA allows finding an appropriate base (PCA base) since it decomposes a data set according to its variance. The PCA base consists of the eigenvectors (PCA vectors) sorted in descending order of the explained variance (eigenvalues) of the original data set. The most dominant movements are the ones explaining the largest variance and are reflected by the first PCA vectors. A PCA base has also been used to detect differences due to footwear conditions [3], [4]. The selection of the base or principal modes of the PCA based on only the first few PCA base vectors and capturing about 95% of the variance, is disputable [14]. If one is interested in extracting differences of movements one will find them in higher order PCA base vectors [3], [4]. The dominant movements, the ones reflected by the lower ordered PCA vectors, account for the highest variance and are not necessarily the ones that are sensitive to external influences to the body, such as wearing different shoes. To investigate how a movement changes when wearing different shoes, one should select a base that distributes the data with respect to highest separation. The linear SVM uses a margin maximizing discriminant analysis [15]. These discriminants can be used repetitively to span a base with the characteristic to optimally separate between two conditions. The projections on the discriminant can be used to assess whether the differences are significant by calculating the classification rate [11]. One has to be aware that separability and the ability to classify (classifyability) are not the same. Separability refers to the fact that the data of two groups can be separated in the data space, however, this does not guarantee that the discriminant separating the two groups in data space can classify an unknown new data set. Classifyability measures the ability to assign unknown data to the correct group and is therefore more restrictive. In case multiple discriminants are found by a repetitive application of the SVM in the orthogonal complement to the previous SVM discriminants, these discriminants will form a SVM base. It is, however, unavoidable that the projections of the data onto the SVM base vectors are correlated and are therefore not independent.

An emerging pattern recognition approach to study statistical independence between multiple time series is the ICA [16], an algorithm that was developed to solve the blind source separation problem [17]. It is frequently used in neurophysiological studies involving recordings of human brain dynamics [16], [18]. So far, ICA has been tested as a tool to identify independent movement components in normal human gait [19], however it has not been used to discriminate two movements caused by shoe or other exterior interventions. It is expected that ICA applied to kinematic data reveals insight into independent components that discriminate patterns. Because each SVM discriminant separates the same two groups, the projections of the data onto two SVM discriminants always reveal a correlation. This violates the definition of independency. Therefore all SVM discriminants are not independent. The ICA isolates one vector that discriminates the two groups. If any of the other ICA base vectors would discriminate the two groups they would not be independent. Thus the ICA will yield exactly one ICA vector with the ability to classify dichotomous data. The vectors obtained by the ICA do not necessarily form an orthogonal base.

The basic motivation for this work was that PCA is not ideally suited to find a base (modes) that are appropriate for the extraction of the part of the movement that allows classification. The purpose was to develop a method that is suited for separating movement differences in dichotomous kinematic data. The aim was to replace the base vectors, also called modes [3], [4], [14], that were usually found using a PCA by an iterative application of the SVM. The novelty consists in using the criterion of classifyability instead of the criterion of variability to compute base vectors of a subspace (SVM space) that contains the information about classifiable aspects of a movement. Within that subspace one can search for discriminants separating two movement patterns. One discriminant is inherently present by the first SVM discriminant. This discriminant will be compared using the effect size [20] to a discriminant found by an ICA applied in the SVM space. In this manuscript we used, as an example, movements resulting from wearing different shoes which allowed us to develop and test the methodology. It is hypothesized that (1) the classification by most SVM base vectors is higher than just using the PCA base vector with the highest classification rate and (2) that there might be a redundancy with respect to the ability to classify the data in the SVM space. (3) The ICA will yield an ICA base whereby only one ICA base vector, the ICA discriminant, will discriminate between the two conditions.

## Methods

### Subjects and ethics statement

Eleven male, healthy, physically active, recreational athletes with a weekly mileage of at least 25 km participated in this study (23.81±5.51 years, 176.38±4.93 cm, 72.25±6.18 kg, mean±SD). Subjects had to be free from lower extremity injury at least six months prior to the study. All subjects gave their written informed consent in accordance with the University of Calgary's policy on research using human subjects. The study protocol was approved by the Conjoint Heath Research Ethics Board at the University of Calgary.

### Experimental protocol

Two different shoe conditions with almost identical construction were tested. The only difference between the shoes was the midsole material in the heel part of the shoe. The first shoe had a viscoelastic heel (stiffness: 167N/mm, maximal deformation: 31.7%, energy loss: 49.5%) and the second shoe had a fully elastic midsole material in the heel area (stiffness: 133N/mm, maximal deformation: 42.4%, energy loss: 26.1%).

Kinematic data were collected using 13 retro reflective markers that were attached to the pelvis and the right lower extremity. A motion capture system with eight infrared cameras (Motion Analysis, CA) was used to collect kinematic data with a sampling frequency of 240 Hz. Each subject performed running trials on a 30 m indoor runway. A force platform embedded in the running lane was used to identify time of initial ground contact and toe off. For each subject, twenty trials were collected in each shoe condition with an average speed of 4±0.2 ms^−1^. The testing order of the shoes was randomized throughout the study and each subject was given a familiarization period of ten minutes prior to data collection as well as a five minute rest period between the two conditions in order to avoid fatigue.

### Data preparation

Markers were identified and tracked using EVaRT Real Time (Version 5.0.4, Motion Analysis, CA). Kinematic data were filtered using a low pass fourth order Butterworth filter with a cutoff frequency of 12 Hz. Kinematic data were only analyzed for the stance phase of the right leg which was identified using the force platform data. A level of 15 N was used as a threshold for the vertical ground reaction force to identify the time points of initial ground contact and toe off. Kinematic data during stance phase were time normalized and resampled using MATLAB's resample function to obtain 101 equidistant time points. For the combination of different trials and subjects kinematic data were shifted to the center of the pelvis marker in the transversal plane and to the floor level in the vertical direction. Subsequently, the data of all subjects were normalized to their individual height.

### Organization of kinematic data into a matrix

An input matrix *M* was built that contained all kinematic data from the experiments. The matrix was organized as follows. For every trial, the time dependent 3D coordinates of all 13 markers were joined together in a row vector. The first 101 components of this row vector were the x positions of marker 1, the second 101 components the y positions, the third the z positions, the fourth the x positions of the second marker and so on. This results in a 1×3939 row vector per trial. All trials and conditions of all subjects were placed in individual rows of *M*. Therefore *M* was a matrix of size 440×3939 (440 = 11 subjects ×2 conditions ×20 trials per conditions; 3939 = 101 time points ×13 markers ×3 directions). In order to reduce subject specific differences each variable was whitened within each subject resulting in an input matrix *M*':
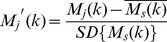
(1)with *j* being the index of the trial, *s* the index of the subject and *k* the index of the 3939 variables. 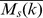 and 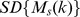 are the mean and standard deviation over the trials and conditions within one subject, respectively. The resulting input matrix *M*' had zero mean and standard deviation of one for every variable.

### Layout of the analysis procedure

**Figure 1:**
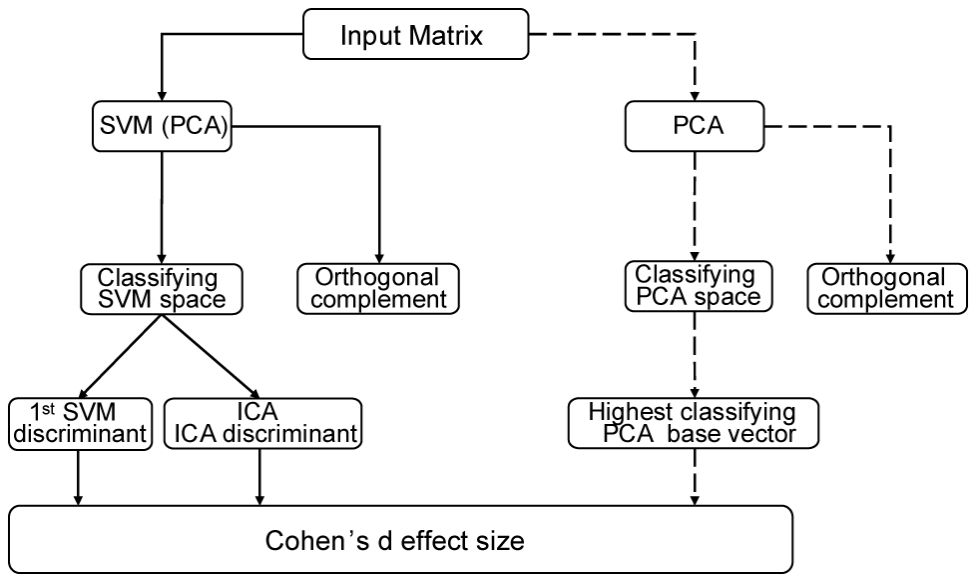
Flow chart of the methodological approach. Flow chart of the decomposition of the input matrix. Subspace identification using an SVM and an ICA is shown in solid black arrows. Decomposition into a PCA space is shown in dashed black arrows and represents the approach used previously by other researchers. For all methods the best classifying base vector was extracted and the Cohen's d effect size was calculated.

The focus of this paper will be on the classifyability and thus, the leave one out method will be used throughout the analysis [21]. The individual steps that will be used to obtain the PCA base, the SVM base and the ICA base are explained below. The PCA base will be used to reproduce and compare the methods published elsewhere [3], [4]. The SVM base will be introduced as an alternative to the PCA base. The SVM base spans a subspace of the data space that is optimally suited to extract the multi-dimensional part of the movement that depended on the shod conditions. The SVM base is a set of vectors that all can classify the two groups. In turn, the orthogonal complement of the SVM space contains the part of the movement that is unaffected by the shod condition and the two shod conditions cannot be classified in this space. An attempt was made to reduce the redundancy of the SVM space by applying an ICA within the SVM space. A flow chart of the basic layout of the analysis is provided in (Figure 1).

### PCA base computation

PCA was applied to the input matrix *M*'. The dimensionality, *n*, of the data space was calculated as *n* = rank{*M*'}. The PCA base vectors were calculated using a singular value decomposition [22]:
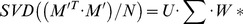
(2)where *M*'*^T^* is the transpose of the matrix *M*' and *N* is the number of trials over all subjects and conditions. The first *n* vectors of *U* represent the PCA base of the data in *M*'. The eigenvalues representing the variance are diagonal elements of 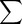.

To obtain the results from the previous approach [11], projections of *M*' onto the PCA base were performed and the classification rate was calculated. The classification rate was calculated with a leave one out method. One subject was removed from the input matrix. The PCA was then performed on the remaining data and for the subject that was removed the rate of correctly assigned trials was calculated. This process was repeated for all subjects and the mean classification rate was calculated. PCA base vectors that yielded significant classification rates were isolated and defined a subspace, the classifying PCA space. The PCA space is therefore subdivided into the classifying PCA space and the non-classifying PCA space. A reconstruction of the movement based on the classifying PCA space only will show the multi-dimensional movement that changes when wearing the two different shoes whereas the reconstruction from the non-classifying space will show the multi-dimensional movement that remains unchanged when changing shod condition. The sum of the two partial movements again represents the original movement.

### SVM base computation

A support vector machine is designed to find the largest margin between two groups. Support vectors within each group are assigned and combined in order to classify a pattern *x* into one of the two groups. The general discriminant function is given by:
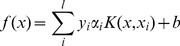
(3)where *l* represents the number of support vectors, *y_i_* the target value of the support vector *x_i_*. The *α_i_* are the weight factors of the support vector *x_i_*. The constant *b* is a bias and *K(x,x_i_)* is the kernel function [21], [23].

Different linear and nonlinear kernels have been used in the literature [21], [23]. We used a linear kernel that allows the calculation of a discriminant vector using equation 4.
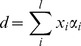
(4)


A linear kernel has the advantage that the discriminant vector is a vector of the data space and therefore linear vector analysis can be applied. The vector *d* is vertical to the plane that separates the data points in the data space. A projection onto this vector will show an accumulation of points on either side of the separating plane and thus allows discriminating points that belong to one or the other group. The bioinformatics toolbox from MATLAB was used to calculate the SVM.

To find the SVM base vectors an iterative procedure was used. The iterations were made along the index m of the SVM_m_-base vectors. The following steps were done whereby each of the 11 subjects with index *s* was sequentially used as the one left out.

Step 1: The SVM, was applied to the matrix *M*', which in this case represents the data from all but one subject (leave one out). A SVM discriminant was calculated.

Step 2: The discriminant was used to assess how many of the trials of the subject with index *s* were correctly assigned to the two shod condition. If 25 out of the 40 trials were correctly assigned then the subject's data were deemed classifiable according to a binomial test (p<0.05).

The percentage of subjects whose data were classifiable was called the classification rate. If the data of 8 of the 11 subjects were classifiable (classification rate 8/11 = 73%) a significant classification rate was achieve based on a binominal test (p<0.05). A discriminant was calculated for each iteration of the leave one out method resulting in 11 discriminants. If a significant classification rate was found the 11 discriminants point almost in the same direction. An overall discriminant was calculated by taking the mean of the 11 discriminants. The overall discriminant was used as the SVM_m_-base vector. The orthogonal complement to the SVM discriminant(s) was then computed in the data space.

The iteration was continued for the next index, *m*, using the vectors in the orthogonal complement to form the new *M*'. The iteration ended when the classification rate dropped below 73%. At this point the SVM space has been obtained with m base vectors.

The vectors that span the last orthogonal complement cannot be organized with the SVM as no classifiable information remained in the data of this subspace. Therefore, we used the PCA to form a base according to the variance in the data. This PCA base allows a reconstruction of that part of the movement that is not contributing to the classification. The joint SVM base and PCA base of the orthogonal complement, together, form an orthogonal base of the data-space. To each one of the base vectors one can assign a fraction of the variance of the raw data by computing the mean of the squared projections of all data sets. Thus one can compute the amount of variance explained by the SVM base and the PCA base of the orthogonal complement.

If the SVM space is multi-dimensional then the data projected on the base vectors are not independent. In that case, an ICA was used to further transform the SVM base into a non-orthogonal ICA base of the SVM space. From the ICA base vectors the one was selected that discriminates between the two conditions.

### ICA base computation

The *runica* algorithm from the open source Matlab toolbox *EEGLab* was used to compute the ICA base of the SVM space [24]. The ICA algorithm yields a number of base vectors that is equal to the number of SVM base vectors.

It is important to note that the ICA base is not anymore an orthogonal base but represent statistical independent vectors which span the SVM space. For detailed information regarding the ICA, the reader is referred to classical ICA literature [16]. The ICA discriminant, usually the first ICA base vector, allows the separation of the two shod conditions.

### Recovering and visualizing the movement

The movement component within any subspace of the kinematic data space, one dimensional or multi-dimensional, can be recalculated as follows: 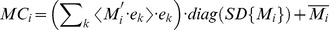
(5)


with 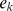 a base vector with unit length, 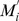 the kinematic pattern of one trial, 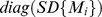 contains the standard deviation across one subject for all the input dimensions and 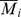
is the mean across trials of one subject. In case there was more than one SVM base vector, the data 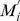 were projected on all SVM base vectors *e_k_* that together formed the classifying subspace (SVM space). The term 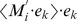 gives the contribution of the base vector k to the movement in the direction of the base vector 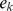. The sum over all contributing base vectors gives the movement component of the whitened data. To retrieve the movement in meter each variable had to be multiplied by the standard deviation over all trials. This is achieved by multiplying the vector 
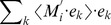
by the diagonal matrix 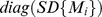. Finally the mean value was added to move the marker positions back to the global coordinate system by adding the mean value 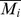.

To recover the movement that is different for the two conditions the e_k_ of the SVM base was used. The movement component was recovered using equation 5 for both conditions and averaged over the different trials within the conditions. A movie of the kinematic data during the stance phase was produced, where the two conditions (red dots representing the viscoelastic and blue dots representing the elastic midsole) where superimposed (Video S1). A time shot where the biggest difference was visible in the movie was produced. The same procedure was applied using the PC base of the orthogonal complement to the SVM space. Again, the movement component was recovered for both conditions and superimposed in a movie (Video S2). The video shows that the blue and red markers moved in synchrony.

The waveforms are the time dependent changes of the marker positions. The waveform of the marker showing the largest changes was derived from the input matrix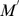. The marker *n* in the dimension *j* is located in the columns.
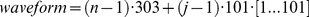
(6)[1...101] represents the range in intervals of 1 normalized time unit. There is one waveform per trial (rows in 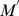). These waveforms indicate the displacement from the mean position of one marker in one direction over the entire stance phase. The waveforms for one or the other shod condition were averaged and the standard deviation was computed for each normalized time point.

### Statistics

A binominal distribution with probability set to 0.5 and α set to 0.05 was used to determine how many trials and subjects were needed to achieve a significant level of the classification. For the leave one out method, the number of correct trials required was based on the total number of trials for one subject, for the mean discriminant the number of correctly classified subjects required was based on the total number of subjects.

Histograms of the projections of the kinematic data onto the classifying PCA base vectors, the SVM base vectors and the classifying ICA base vector that showed the highest classification rates were generated. The histograms were used to assess whether the values were normally distributed (Lilliefors test [25]) for both shoe conditions. If so, the Cohen's d effect size was computed [20].

## Results

### Classification within the PCA space

**Figure 2:**
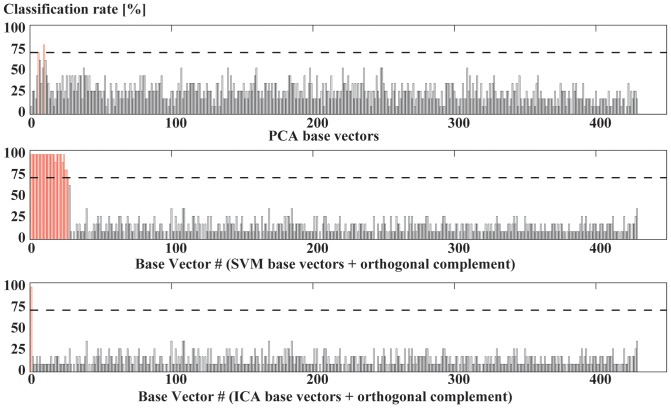
Classification rate of different methods. Classification rate of all base vectors using a PCA (top), a SVM (middle) and an ICA (bottom). Base vectors that allowed for significant classification are shown as gray bars and those that do not classify are shown as black bars.

Significant classification of the projected data onto the principal components was achieved within two principal components. Principal component #6 and #10 allowed for a significant classification rate of 72.7 and 81.8%, respectively (Figure 2). It follows that principal component #10 classifies the data best and is, thus, considered the highest classifying PCA base vector. The explained variance for components #6, and #10 and was 3.3%, and 1.8%, respectively. These PCA vectors form the classifying PCA base. The effect size of PCA base vector #10, showing the highest classification rate, was 1.4.

### Classification within the SVM space

The calculation of the SVM base revealed 27 base vectors that allowed for a significant classification (Figure 2) and span the SVM space. In total 22 base vectors classified 100%, the mean classification rate of the SVM space was 95.78% (SD 9.42%). The explained variance within the SVM space was 2.73%. The first SVM base vector, which already explained 1.65% of the variance, is sufficient for the classification purpose. The effect size of the first SVM base vector was 8.3. The orthogonal complement to the SVM space contains the remaining variance, however, did not allow for any additional classification.

### The ICA discriminant

Applying the ICA to the data in the SVM space yielded a 27 dimensional, non-orthogonal ICA base of the SVM space. The first ICA base vector, ICA discriminant, allowed separating the two shod conditions (Figure 2). The ICA discriminant explained 0.6% of the variance and thus much less compared to the variance explained by the first SVM base vector. The effect size of ICA discriminant was 6.9. The rest of the ICA base vectors did not reach any significant separation and were not further considered.

### Loading of the components and visualization

**Figure 3:**
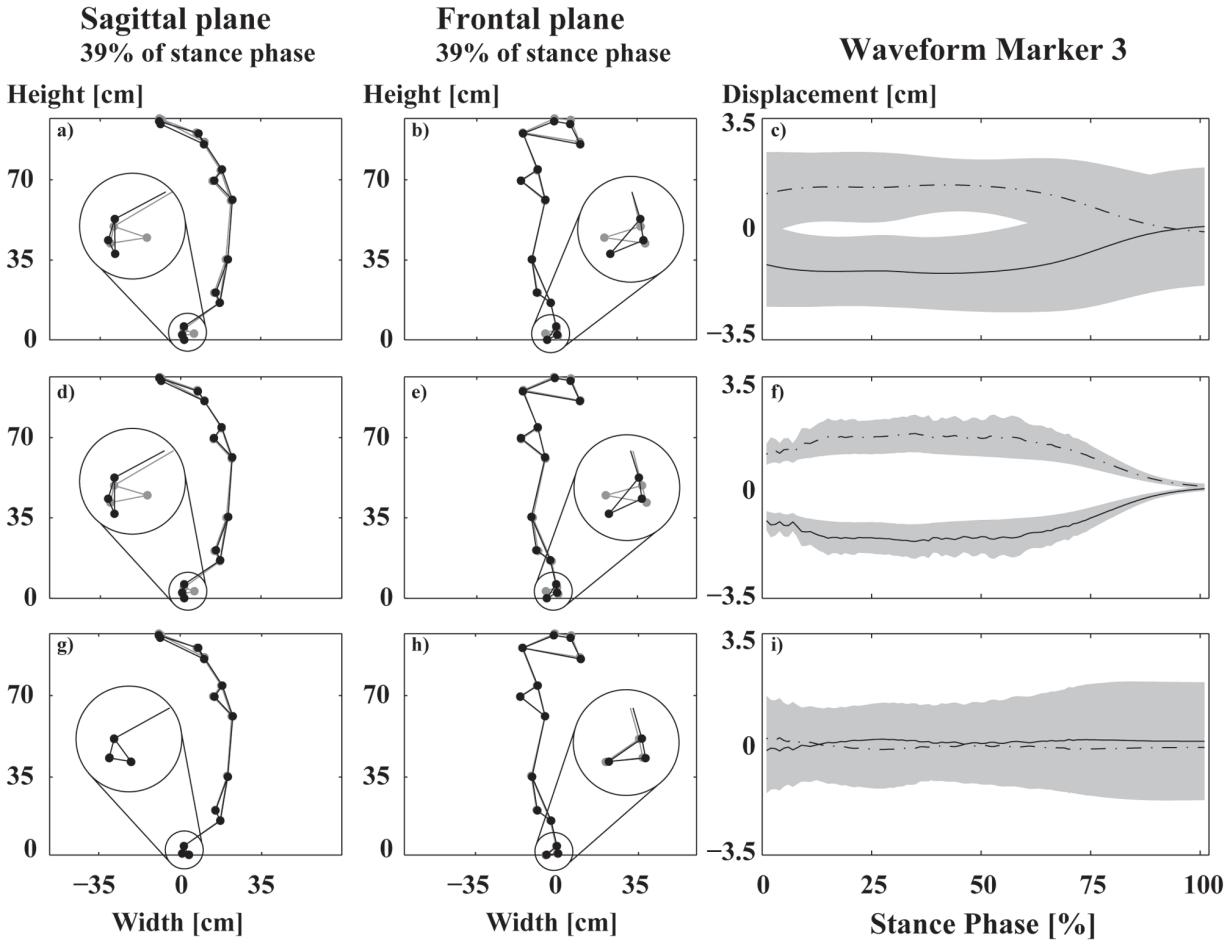
Visualization of movement differences. Stick figures of all markers in the sagittal plane (a,d,g) and the frontal plane (b,e,h). The waveforms of marker three that separated both shoe conditions are shown in the third column (c,f,i). The first row (a-c) shows the result using the classifying PCA base. The second row (d-f) shows the approach using the SVM base. The third row (g-i) shows the orthogonal complement to the SVM base. The differences of the stick figures are visualized using a magnification factor of 5. The black and grey dots in the stick figures represent the viscoelastic and elastic midsole, respectively. The largest changes were found at the ankle (circle). The figures in the third column show the waveforms and their standard deviations (gray shaded area) for the two shod conditions. The dashed line represents the elastic midsole and the solid line the viscoelastic midsole.

Independent of the pattern recognition method chosen, the highest loading (biggest absolute value of the discriminant vector) on the first SVM base vector occurred at line 847 of the matrix *M*'. This corresponds to the third marker (attached to the shoe) in vertical direction at time point 39 (39% of the stance phase). At this time point static images of the marker positions were visualized in the sagittal (Figure 3 a,d,g) and frontal plane (Figure 3 b,e,h) for different subspaces. When plotting the corresponding waveforms and their standard deviation for the classifying PCA base and the SVM base, the results show that waveform differences were larger for the SVM base than for the classifying PCA base. Thus, the SVM base leads to a more distinct separation compared to classifying PCA base (Figure 3 c and f). Furthermore, the orthogonal complement to the SVM space does not contain any visible differences anymore (Figure 3 i).

## Discussion

The discrimination of aspects of the human movement and the classification is of central importance to biomechanical studies. The SVM decomposition method is focused on revealing movement difference and represents an alternative to a PCA method that focuses on finding principal movements. The first principal movements may not be the ones that differ when applying external interventions. To our knowledge, there is no similar approach that we could compare our method to. The method has to be seen as an expansion of the methods previously used [3], [4], [11]–[13]. The results show that if one stops after the first SVM discriminant is found one might be able to assign a movement to one or the other group but parts of the movement that differs between the shod conditions remains undetected. To visualize the difference caused to the movement by the shod conditions it is important not to stop the analysis before all SVM base vectors are available and thus the subspaces, the one containing the discriminatory information (SVM space) and the one who accounts for the remaining variance (orthogonal complement to SVM space) of the data, are known. However, it is obvious that the different bases are not identical, each one is worth considering and the base vectors have to be interpreted and used according to the question of interest.

The PCA base is very important if one wants to analyze the main features of a movement. It is common practice to use only the first few PCA base vectors and one can then reproduce the movement and regain most (95% or more) of the variance. Thus, this corresponds to a de-noising process and to a process reducing the dimensionality. In addition, the first PCA base vectors were viewed as descriptors of principal movements representing body segments moving in a correlated way. This dissection into principal movements [3], [4], [13] is important for the visualization and assessment of different aspects of the movement e.g. discriminating the leg movement from the movement of the center of mass. However, as seen in Figure 2, the information about differences between two conditions is only available in higher order PCA base vectors. One is never sure whether one has ideally separated the PCA base vectors into those that do contain all the information that can be used for classification purposes. In light of the current method, the PCA approach for separating kinematic data [3], [11] can be significantly improved using the SVM and ICA methods, confirming our first hypothesis. However, the SVM base shows that there were 27 SVM base vectors. All of them were able to differentiate the two shod conditions. In the PCA decomposition (Figure 2), the classifying PCA base vectors that are able to classify occurred in an unordered sequence and had to be searched for after the PCA was done. In contrast, the SVM decomposition of the data space yielded all SVM base vectors in an ordered manner, ranking them in accordance to the variance. The SVM decomposition is therefore much more efficient. From the classification point of view, one has achieved the goal with the first SVM base vector which yields the best classification based on maximizing the margin between the data of the two conditions. That is where SVM analysis normally ends [11]. However, this does not tell us whether there are multiple vectors with different loadings that are equally able to classify the data. It can be seen in figure 2 that there is a multitude of SVM base vectors which also allow 100% correct classification, indicating that the SVM does not identify one unique discriminant. This redundancy in the SVM space confirms our second hypothesis. Nevertheless, the first SVM base vector is not sufficient to describe the discriminating movement. We used the SVM space to reconstruct (visualize) the whole aspect of the movement that contains the effect of shoe differences on the movement and we used the orthogonal complement to reconstruct the part of the movement that did not change when wearing different shoes. Using this technique one can visualize aspects of the movement that represent only a few percent (2.73% in the present case) of the variance of the movement but reveal the differences that occur when wearing different shoes and allow the classification. Watching the attached video allows the user to visually get a better understanding of how the movement changes when wearing different shoes.

As mentioned above, the SVM base contains a redundancy with respect to the classifyability. This redundancy becomes obvious when one looks at the projections of the kinematic data onto the SVM base vectors. These projections are correlated because they are separating the same dichotomous data. Thus we do not know whether there are independent movement patterns that are still a consequence of the shod conditions. For this reason an ICA was applied to the data in the SVM space. The ICA is an unsupervised pattern recognition tool, however, the ICA algorithm contains no criterion that requires the optimal separation of the shod conditions. Thus forming the SVM space that contains the information about the shod conditions first is necessary before the ICA can selects one direction in SVM space. By limiting the ICA to the SVM space, the irrelevant aspect of the orthogonal complement is not contributing disturbances to the ICA algorithm.

At the end of the analysis one could select the classifying PCA base vector (effect size 1.4), the first SVM base vector (effect size 8.3) or the ICA discriminant (effect size 6.9) for the purpose of classifying kinematic data of new subjects. Based on the effect size, however, we would recommend using the first SVM base vector.

The fact that the effect size of the ICA discriminant was smaller than the one of the first SVM base vector points to remaining open questions and limitations that require further methodological improvements.

Currently we are still skeptical of the uniqueness of the ICA discriminant because the effect size and the variability explained by the ICA discriminant are both smaller than for the first SVM base vector. One may have to introduce an additional condition e.g. the effect size, into the ICA algorithm. We have no means to test the uniqueness of the ICA discriminant with the leave one out approach described in the layout of the analysis and with the current set of data. One would have to return to a pure separability analysis, computing a SVM base using separability as a criterion for accepting a SVM discriminant and then testing the ICA discriminant. While trying out alternative methods we also used another SVM software (results not shown) and found that the dimensionality of the SVM space depended on the parameters and the computational procedures used in different SVM software packages [26]. For instance, if a reduction of the margin is accepted for the SVM computation the SVM space alters its dimensionality and can eventually collapse to a single dimension. A systematic investigation of all possible alternatives was beyond the scope of the current work. In principle, one could interpret the dimensionality of the SVM space as a measure for the complexity of a movement. However, with the above mentioned uncertainties in the calculation of the dimension it is at this stage not advisable. These methodological differences do not alter the principal insight we have obtained with respect to the differences of the movements caused by the shod conditions, however, one has to remain cautious when interpreting minute aspects that may depend on the computational procedures.

## Conclusion

The current work has shown that a data space of dichotomous kinematic data can be decomposed into two subspaces by using an iterative decomposition into discriminants obtained by a repetitive application of a SVM. The SVM space contains all the information that discriminates the aspects of the movement that is used for the classification of the two shod conditions. One can show how much of the variance of the movement can be assigned to the movement described by the SVM base compared to the variance explained by the orthogonal complement. It was shown that the SVM base contains some redundancy and a single ICA discriminant was found by applying an ICA to the kinematic data when they are represented in the SVM space. We therefore conclude that a combination of SVM (to subdivide the whole space into two subspaces) and ICA is best suited to find a discriminant, either the first SVM base vector or the ICA discriminant, for the classification of dichotomous kinematic data. Based on the effect size, we would favor the first SVM base vector. One of our future projects is to improve the ICA algorithm by including a criterion that optimizes the effect size. The reconstruction and visualization of the data within the SVM space represents a means to visualize the part of the movement that allowed the classification to be done.

## Supporting Information

Video S1 (WMV)

Video S2 (WMV)
